# Mitochondrial transfer-driven immune evasion in the tumor microenvironment

**DOI:** 10.1093/intimm/dxag027

**Published:** 2026-05-29

**Authors:** Li Zhu, Yosuke Togashi

**Affiliations:** Department of Tumor Microenvironment, Graduate School of Medicine, Dentistry and Pharmaceutical Sciences, Okayama University, Okayama 700-8558, Japan; Department of Tumor Microenvironment, Graduate School of Medicine, Dentistry and Pharmaceutical Sciences, Okayama University, Okayama 700-8558, Japan; Advanced Research Field, Institute for Emerging Medical Innovation, Okayama University Hospital, Okayama 700-8558, Japan; Department of Respiratory Medicine, Okayama University Hospital, Okayama 700-8558, Japan; Faculty of Medicine, Kindai University, Osaka 589-8511, Japan

**Keywords:** immune checkpoint blockade, mitochondrial transfer, T-cell exhaustion, tumor-infiltrating lymphocytes

## Abstract

The tumor microenvironment (TME) is a complex landscape where metabolic interactions significantly dictate antitumor immunity. Immune evasion in cancer is typically discussed in terms of inhibitory receptors and ligands, suppressive cytokines, defective antigen presentation, and metabolic competition. However, recent evidence reveals that intercellular mitochondrial transfer adds a new mechanism of immune evasion in the TME. The mitochondrial fitness of T cells is central to sustained effector function, memory formation, and responsiveness to immune-checkpoint blockade. Tumor cells can act as pathogenic mitochondrial donors, transferring functional or dysfunctional mitochondria to neighboring T cells via tunneling nanotubes and extracellular vesicles. This process involves a mitophagy imbalance that leads to the homoplasmic replacement of endogenous mitochondria, thereby driving T-cell senescence, impairing memory formation and long-term antitumor function, and ultimately weakening cancer immunosurveillance. Overall, mitochondrial transfer should be considered a new part of the tumor immune evasion framework. It also provides new therapeutic opportunities for improving cancer immunotherapy.

## Teaser

Mitochondrial transfer suppresses anti-tumor responses

## Introduction

Immune evasion is a core feature of cancer progression and a major reason why immunotherapy does not work equally well in all patients (1–6). Tumor cells escape immune control through several mechanisms. They can reduce antigen recognition, express inhibitory ligands, and build an immunosuppressive tumor microenvironment (TME) (1, 4–6). In this setting, inhibitory immune-checkpoint pathways, especially CTLA-4 and PD-1–PD-L1/PD-L2, became major therapeutic targets. As a result, immune-checkpoint inhibitors (ICIs) showed that endogenous antitumor T cells can be re-activated in patients with cancer (7–9). However, the durable benefit is limited to a subset of patients. Many patients show primary resistance, and others later develop acquired resistance after an initial response (2, 3). These findings suggest that checkpoint signaling alone does not fully explain long-term immune failure in cancer.

One important part of this problem is metabolic stress in the TME (10, 11). Tumor-infiltrating lymphocytes (TILs) are exposed to hypoxia, nutrient restriction, oxidative stress, and persistent antigen stimulation (11–14). These conditions gradually reduce T-cell fitness and promote dysfunctional states (11–14). In this process, mitochondria are especially important because they support oxidative phosphorylation (OXPHOS), adenosine triphosphate (ATP) production, redox balance, and stress adaptation (15, 16). Therefore, loss of mitochondrial integrity in T cells has consequences that go beyond reduced bioenergetic efficiency. It impairs cytotoxic function, weakens memory potential, and limits the durability of responses to PD-1 blockade (17–19). For this reason, mitochondrial fitness should be considered a key determinant of antitumor T-cell function.

Intercellular mitochondrial transfer adds new insight to this framework (20–24). Early studies mainly focused on transfer from stromal or host cells to tumor cells, and this was understood as a way for tumor cells to restore respiration, survive metabolic stress, and resist therapy (20, 21, 25, 26). More recent work expanded this view and showed that mitochondrial transfer in the TME can also involve immune cells, especially T cells (20, 21, 27–29). This shift suggests that tumors may do more than expose lymphocytes to an unfavorable metabolic environment. They may directly alter T-cell mitochondrial identity through organelle transfer (27). Here, we discuss this emerging model of immune evasion. We begin by outlining why mitochondrial fitness is critical for antitumor T-cell function. We then examine tumor-to-T-cell mitochondrial transfer, including its routes, persistence, and consequences for T-cell fate, and finally consider its broader impact on the TME and its therapeutic implications. The proposed model of tumor-to-T-cell mitochondrial transfer in the TME is summarized in Figure 1.

**Figure 1.**
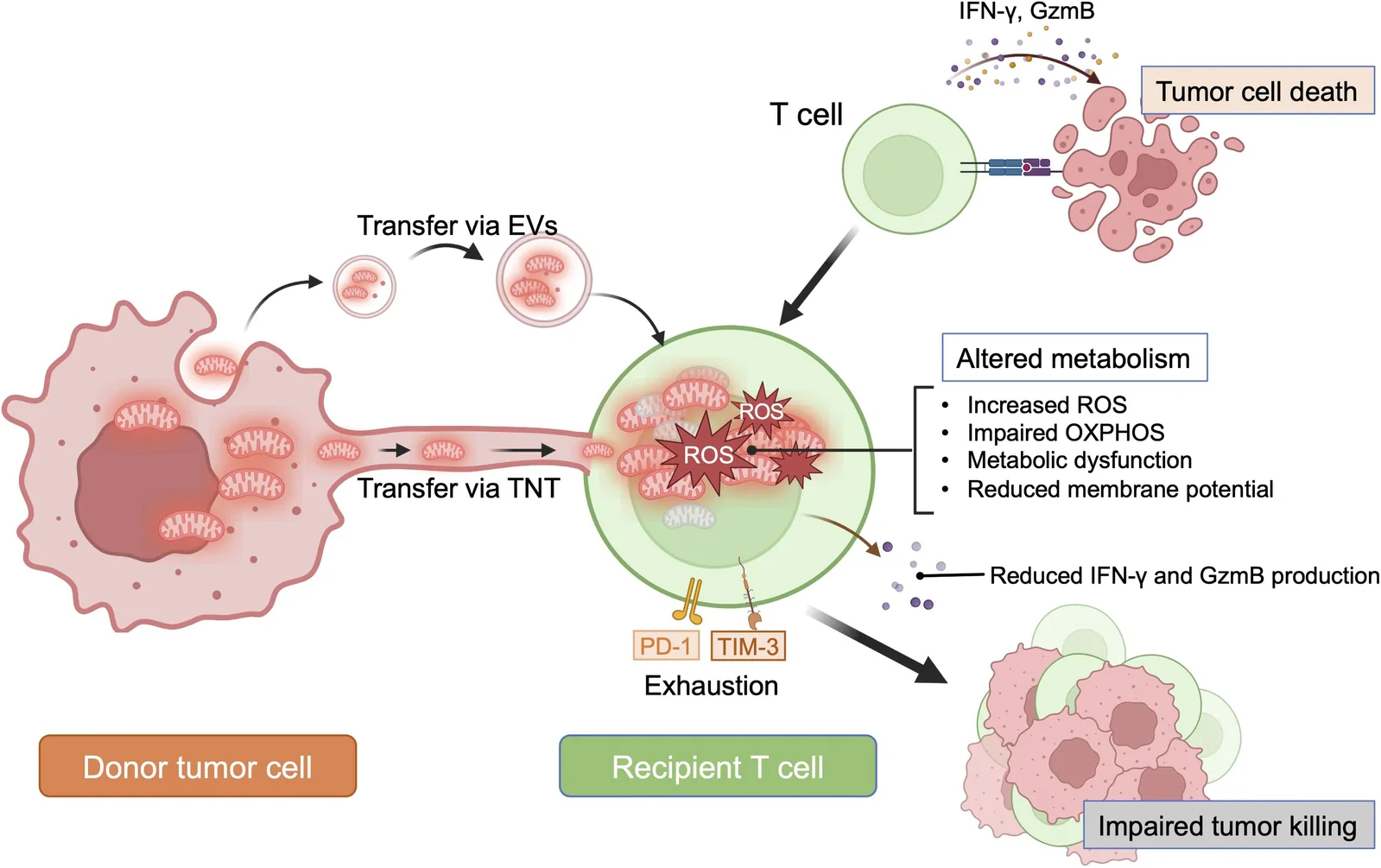
Overview of tumor-to-T-cell mitochondrial transfer in the tumor microenvironment. Tumor cells may donate mitochondria to neighboring T cells via EVs and TNTs. In recipient T cells, transferred tumor-derived mitochondria are proposed to accumulate and alter cellular metabolism, with increased ROS, impaired OXPHOS, reduced membrane potential, and greater dependence on glycolysis. These changes are associated with increased expression of exhaustion-related surface molecules together with reduced effector function, as indicated by lower IFN-γ and GzmB release and impaired killing capacity. Red mitochondria indicate transferred tumor-derived mitochondria, whereas pale mitochondria indicate pre-existing mitochondria in the recipient T cell. EVs, extracellular vesicles; TNTs, tunneling nanotubes; ROS, reactive oxygen species; OXPHOS, oxidative phosphorylation; PD-1, programmed cell death protein 1; TIM-3, T-cell immunoglobulin and mucin-domain containing-3; IFN-γ, interferon-γ; GzmB, granzyme B. Figure created with BioRender.

## Mitochondrial fitness as a basis for durable antitumor T-cell function

T-cell mitochondrial dysfunction is already recognized as a major barrier to durable antitumor immunity (17–19). A more recent advance is the recognition that mitochondrial transfer now partially offers a direct upstream mechanism that may help explain how this dysfunction is imposed in the TME (27). In T cells, mitochondria are not only sources of ATP but also coordinate bioenergetic adaptation, reactive oxygen species (ROS) signaling, and differentiation programs (17, 18, 23). Early after activation, T cells increase glycolysis, but mitochondrial metabolism remains essential for full activation and effector programming (14, 17, 18). In particular, mitochondrial ROS are required for NFAT activation and IL-2 induction, which places mitochondria within the signaling machinery of T-cell activation (30, 31).

This becomes even more important when durable T-cell responses are considered. Glycolysis supports rapid effector activity, but by itself it is insufficient to sustain long-term immunity (14, 17, 18). Memory formation and long-term survival require strong mitochondrial respiratory capacity, and memory T cells have high spare respiratory capacity that supports persistence under stress (32). In tumors, this dependence is especially important because T cells are exposed to persistent antigen stimulation and a metabolically hostile environment (11–14). Reviews and primary studies have shown that hypoxia, low nutrient availability, and chronic stimulation in the TME drive metabolic insufficiency in TILs and weaken antitumor function (11, 33–35). Exhaustion is often described through transcriptional hierarchies and checkpoint receptor expression, yet it is also a metabolic state (12, 36). PD-1 signaling suppresses mitochondrial as well as glycolytic programs, and exhausted T cells show defects in mitochondrial biogenesis, respiratory competence, and organelle dynamics (31, 33, 34, 37).

In mouse tumors and human TILs, repression of mitochondrial biogenesis, disturbed mitochondrial dynamics, and reduced mitophagy are closely linked to metabolic insufficiency, loss of effector function, and progression toward terminally exhausted states (31, 33–35). Mitochondrial dysfunction in T cells can also promote senescence-like features and reduce long-term survival (38). These observations support that in tumors, mitochondrial fitness is one of the core determinants of whether T cells can remain functional, maintain memory potential, and respond durably to PD-1 blockade (31–34). If mitochondrial integrity is already a limiting factor for TIL function, then any tumor-derived process that changes mitochondrial quality in recipient T cells can amplify immune resistance even without altering antigen specificity or checkpoint receptor abundance directly.

## Tumor-to-T-cell mitochondrial transfer as a mechanism of immune evasion

### Mitochondrial DNA mutation and mitochondrial transfer

In general, mitochondrial DNA (mtDNA) is intrinsically vulnerable to damage and mutation. It lies close to the electron transport chain, is exposed to ROS, has limited repair capacity, and lacks histone protection (39). These features make mtDNA more prone to mutation than nuclear DNA (39, 40). mtDNA variation is common in tumors and can influence mitochondrial function, metabolic state, and tumor behavior (40–43). In the current key study, mtDNA sequencing of clinical specimens identified mtDNA mutations in TILs from a subset of patients, and many of these mutations were identical to those found in paired tumor cells. These shared mutations were caused by mitochondrial transfer. These findings support the view that tumor-derived mitochondrial genetic material can be detected within lymphocytes at the tumor site itself (27).

### Routes of tumor-to-T-cell mitochondrial transfer

Current evidence supports two main routes for mitochondrial transfer from tumor cells to T cells: direct transfer through tunneling nanotubes (TNTs) and indirect transfer through small extracellular vesicles (EVs) (27, 44). In this study, transfer was reduced by cytochalasin B, which inhibits TNT formation, and it was also reduced when donor and recipient cells were physically separated. Transfer was further reduced by GW4869, which inhibits the release of small EVs. These findings support a model in which both contact-dependent and contact-independent pathways contribute to tumor-to-T-cell mitochondrial transfer.

The TNT route is consistent with earlier work showing that nanotube-based mitochondrial trafficking can occur between tumor cells and immune cells (44). That earlier study mainly highlighted the transfer from immune cells to tumor cells. Still, it established an important principle: direct membrane bridges can support mitochondrial exchange at tumor-immune interfaces and can shape antitumor immunity (44). It also fits with the broader mitochondrial-transfer literature, which places TNTs among the major contact-dependent routes for organelle exchange (20–24). Stress conditions that are common in tumors, including hypoxia, ROS, low pH, and inflammatory signals, have also been linked to TNT formation (45–48).

The EV route provides a way for tumor-derived mitochondrial material to reach T cells without sustained cell–cell contact (49–51). In the current key study, inhibition of small-EV release reduced transfer, and purified EV fractions smaller than 200 nm contained mitochondrial protein together with EV markers (27). This supports the idea that small EVs can carry mitochondrial cargo, including mtDNA and mitochondrial proteins, from tumor cells to T cells. At present, free mitochondria and other less defined contact-independent routes remain possible in principle, but they are not yet supported as the main routes in tumor-to-T-cell transfer (52). The route of transfer determines how often, how far, and under what conditions tumor cells may distribute dysfunctional mitochondrial influence to T cells in the TME (20–24).

### Selective persistence and mitochondrial hijack in recipient T cells

A major mechanistic question arises after tumor-derived mitochondria enter T cells: why are these mitochondria not simply eliminated after transfer? Current evidence suggests that the answer lies in asymmetric mitochondrial quality control within recipient T cells (27). In the TME, ROS are abundant, and damaged mitochondria are normally removed through mitophagy (53, 54). This process is therefore expected to limit the persistence of dysfunctional mitochondria in recipient cells. In the current key study, endogenous mitochondria in recipient T cells were reduced during co-culture with tumor cells, and this reduction was blocked by antioxidant treatment or by mitophagy inhibition (27). These findings support the view that recipient T cells normally clear damaged mitochondria through ROS-linked mitophagy (27, 54).

The same study also suggests why tumor-derived mitochondria may escape this clearance (27). Tumor-derived mitochondria were found to persist in recipient T cells even under ROS-rich conditions, and this persistence was linked to co-transfer of USP30, a mitochondria-associated deubiquitinase that opposes Parkin-mediated mitophagy (27). More broadly, USP30 is already known to antagonize mitophagy by removing ubiquitin signals from mitochondria (55, 56). This makes it a plausible mediator of post-transfer persistence. In this model, endogenous T-cell mitochondria, which lack the same protection, are preferentially removed, while tumor-derived mitochondria gain a selective advantage inside the recipient cell.

This asymmetric clearance partially helps explain why tumor-to-T-cell transfer can progress beyond a simple exchange event. In some recipient T cells, tumor-derived mitochondria do not remain a minor added population. Instead, they can become dominant and even approach near-complete replacement of the endogenous mitochondrial pool. This phenomenon has been described as homoplasmic replacement or mitochondrial hijack (27, 57). A small amount of transferred mitochondrial material would not be expected to alter T-cell behavior in a major way. By contrast, selective persistence followed by partial or near-complete replacement provides a direct mechanism by which tumor cells can impose dysfunctional mitochondrial inheritance on TILs (27).

### T-cell fate after mitochondrial hijack

In the current key study, T cells that acquired tumor-derived, mtDNA-mutated mitochondria showed impaired OXPHOS, reduced membrane potential, increased ROS, and greater dependence on glycolysis (27). These changes were not isolated metabolic defects. They were accompanied by reduced proliferation, impaired activation, and loss of memory-associated features. In the same work, recipient T cells also showed increased β-galactosidase activity and higher expression of senescence-related markers, which placed mitochondrial transfer within a broader program of T-cell dysfunction rather than a narrow bioenergetic abnormality (27).

These findings fit with the broader literature on T-cell exhaustion and mitochondrial failure (11–14, 17–19). Exhausted T cells are not defined only by checkpoint receptor expression. They also show impaired mitochondrial biogenesis, disturbed mitochondrial dynamics, and reduced respiratory fitness (31–34). These changes limit cytokine production, proliferation, and the ability to maintain memory-like potential (31–34). Work in chronic infection and cancer has also shown that the TCF1-positive progenitor-like compartment is important for the proliferative burst after PD-1 blockade, while more terminally exhausted cells are marked by features such as high TIM-3 and low TCF1 (58–60). In the mitochondrial-transfer model, tumor-derived mtDNA-mutated mitochondria pushed recipient T cells away from long-lived, memory-associated states and toward a more terminally dysfunctional phenotype (27). This link connects organelle transfer to a known hierarchy of T-cell fate. It suggests that mitochondrial transfer can move T cells away from the compartment that is most capable of responding to checkpoint therapy (27, 58–60).

This is also where mitochondrial transfer becomes directly relevant to immunotherapy. In the same study, tumor mtDNA mutation status was associated with poorer outcome after PD-1 blockade in patients with melanoma or non-small-cell lung cancer (27). It showed that mitochondrial dysfunction in T cells impaired antitumor immunity *in vivo* and reduced the benefit of PD-1 blockade. These data support that some tumors may resist checkpoint therapy not only by sustaining inhibitory ligands or suppressive signals, but also by corrupting the mitochondrial program that T cells need for reinvigoration and durable control (27, 33). In a separate study, transfer from bone marrow stromal cells improved T-cell metabolic fitness and antitumor efficacy (61). This contrast suggests that the consequence of mitochondrial transfer depends on donor identity, mitochondrial quality, and the state of the recipient cell. In tumors, the most damaging setting appears to be the combination of donor tumor cells, dysfunctional donor mitochondria, and recipient T cells that are already under chronic metabolic stress (20–24).

### Tumor-derived mitochondrial transfer may reshape the TME

Although the T-cell axis is the main focus of this review, tumor-derived mitochondrial transfer may have broader effects in the TME. Recent work has shown that tumor cells can donate mitochondria to fibroblasts and thereby change stromal cell state. In the current key study on this topic, tumor cells from several tumor types transferred mitochondria to fibroblasts in co-culture and in xenograft tumors. This process depended on the mitochondrial trafficking protein MIRO2 in donor tumor cells and led to fibroblast differentiation toward a cancer-associated fibroblast (CAF) state with both myofibroblastic and inflammatory features. The recipient fibroblasts showed increased OXPHOS, ATP production, and ROS, and they secreted factors that supported tumor growth, migration, angiogenesis, and matrix remodeling (62). Importantly, this stroma-focused study did not evaluate mtDNA status, distinguishing it from the aforementioned T-cell study (27, 62). Instead, it mainly linked the acquisition of tumor-cell-derived functional mitochondria to metabolic activation and CAF-like reprogramming in recipient fibroblasts. Thus, the effects of transferring mutant mitochondria should be investigated as a key focus of the future research.

This stromal effect is relevant to cancer immunology even though it is not a direct immune-cell phenotype (63). CAFs regulate matrix deposition and remodeling, engage in reciprocal signaling with tumor and immune cells, and can support angiogenesis and spatial barriers to T-cell entry (64–66). These stromal changes can promote immune exclusion and can worsen T-cell dysfunction indirectly (65, 67). In this setting, tumor-derived mitochondrial transfer may act at two levels at the same time. It may impair antitumor T cells directly, and it may also create a more permissive niche through fibroblast reprogramming (27, 62).

At present, this donor program is best supported in T cells and fibroblasts (27, 62). Whether analogous mitochondrial exchange also reshapes other stromal or myeloid compartments remains less clear. Myeloid cells are abundant in many tumors, especially in macrophage-rich microenvironments (68–70), and it is therefore reasonable to ask whether they also participate in this mitochondrial network, either as intermediary carriers or as recipient cells reprogrammed by tumor-derived organelles. This possibility remains incompletely defined at present.

## Therapeutic opportunities

Current evidence suggests that mitochondrial-transfer biology may be therapeutically actionable at several steps. One option is to interfere with contact-dependent transfer by limiting TNT formation (44). Preclinical work has shown that inhibition of nanotube-dependent mitochondrial trafficking can reduce tumor-promoting organelle exchange and can improve the effect of PD-1 blockade in experimental systems (44). Another option is to limit contact-independent transfer by reducing EV release (49–51). In the current tumor-to-T-cell model, inhibition of small-EV release reduced mitochondrial transfer, improved antitumor immunity, and increased the benefit of PD-1 blockade *in vivo* (27).

Another opportunity lies in modulating mitochondrial fitness more broadly (17–19). Metformin improved antitumor immunity and potentiated PD-1 blockade in preclinical work through reduction of tumor hypoxia (71). Bezafibrate-based mitochondrial support showed synergy with PD-1 blockade in preclinical studies and encouraging metabolic effects in an early clinical study (72, 73). Spermidine also improved antitumor immunity in mice by enhancing mitochondrial function (74). Together, these studies show that mitochondrial state can be manipulated therapeutically. Still, they do not yet show that pathological mitochondrial transfer can be blocked with high specificity in patients.

## Conclusions

Intercellular mitochondrial transfer has added a new insight to current models of tumor immune evasion. The most important recent advance is the recognition that tumor cells can act as mitochondrial donors to TILs. Mitochondrial transfer is not simply a form of metabolic exchange. It can reshape T-cell fate at the organelle level. Tumor-derived mitochondria can persist in recipient T cells, promote homoplasmic replacement or mitochondrial hijack, and drive a coherent program of mitochondrial dysfunction, senescence, memory impairment, and terminal exhaustion. These changes weaken antitumor immunity and reduce the durability of response to PD-1 blockade. Together, these findings support a broader view in which mitochondrial transfer should be considered part of the immune-evasion framework of the TME, alongside inhibitory ligands, suppressive cytokines, and metabolic competition.

However, it is still unclear how generalizable tumor-to-T-cell mitochondrial hijacking is across tumor types, which transfer routes dominate *in vivo* under different conditions, and how selectively harmful donor-to-immune transfer can be blocked without impairing beneficial mitochondrial support to immune cells. These issues are central for future translational work. If they can be resolved, mitochondrial-transfer biology may provide not only a clearer explanation for durable immune failure in cancer, but also a new set of therapeutic strategies to preserve T-cell fitness and improve the efficacy of cancer immunotherapy.
